# Evaluation of single-use bioreactors for perfusion processes

**DOI:** 10.1186/1753-6561-7-S6-P101

**Published:** 2013-12-04

**Authors:** Aurore Polès-Lahille, Flavien Thuet, David Balbuena, Sébastien Ribault

**Affiliations:** 1 Merck Biodevelopment, Martillac, France, 33650

## Introduction

Single-Use Bioreactors are now commonly used for Process Development activities, as seeding bioreactors or to produce Drug Substances. The advantages of this equipment have been well demonstrated over the last years on batch/fed-batch processes. Continuous processes were widely applied in the past to increase the overall productivity of small bioreactors or for sensitive molecule production. The process control, contamination risk and complexity were the main concerns of this operation mode. However, the bioprocessing trends and technology evolution led to reconsidering the perfusion processes. The aim of this study was to combine standard single-use bioreactors with different perfusion technologies and to compare productivity and molecule quality.

## Methods

A CHO cell line producing a mAb was thawed and amplified in shake flasks using Cellvento™ CHO-100 medium. When a sufficient amount of cells was reached, 2 Mobius^® ^CellReady 3L bioreactors were launched in parallel: one in batch mode and one in perfusion mode using Cellvento™ CHO-100 medium. Two perfusion technologies were assessed: the Fibra-Cel^® ^Disks (Eppendorf) and the Alternative Tangential Flow (Refine) ones. The Mobius^® ^CellReady 3L bioreactor was not modified to perform perfusion processes aseptically transferred into a Mobius^® ^CellReady 3L bioreactor through the probe port. Regarding the ATF™ technology, an ATF-2 system was first washed with water then autoclaved and welded to the harvest line of a Mobius^® ^CellReady 3L bioreactor. The bioreactor conditions were 37°C with pH maintained between 6.80- and 7.10. The Dissolved Oxygen set point was 50% and stirrer speed 104 rpm. The viable cell density, viability, metabolism and titers were measured at least daily. The perfusion was initiated at 0.5 vvm when the lactate was above 0.5 g/L and increased daily based on glucose and lactate levels up to 1 vvm for the Fibra-Cel ^® ^technology and up to 2 vvm for the ATF™ one. In order to increase the oxygen transfer at high cell density, a decision tree was applied. For the Fibra-Cel^® ^technology, the mAb was collected in harvest bags welded to a side port while for the ATF™, the molecule remained inside the Mobius^® ^CellReady 3L bioreactor with the use of a 50 kDa hollow fiber. In order to measure the quality of the mAb produced, samples were collected on day 7, day 10 and the last bioreactor day. Titers and HCP levels were directly measured on harvest while SE-HPLC and cIEF were performed on ProSep^® ^Ultra Plus eluates.

## Results

**Figure 1:**
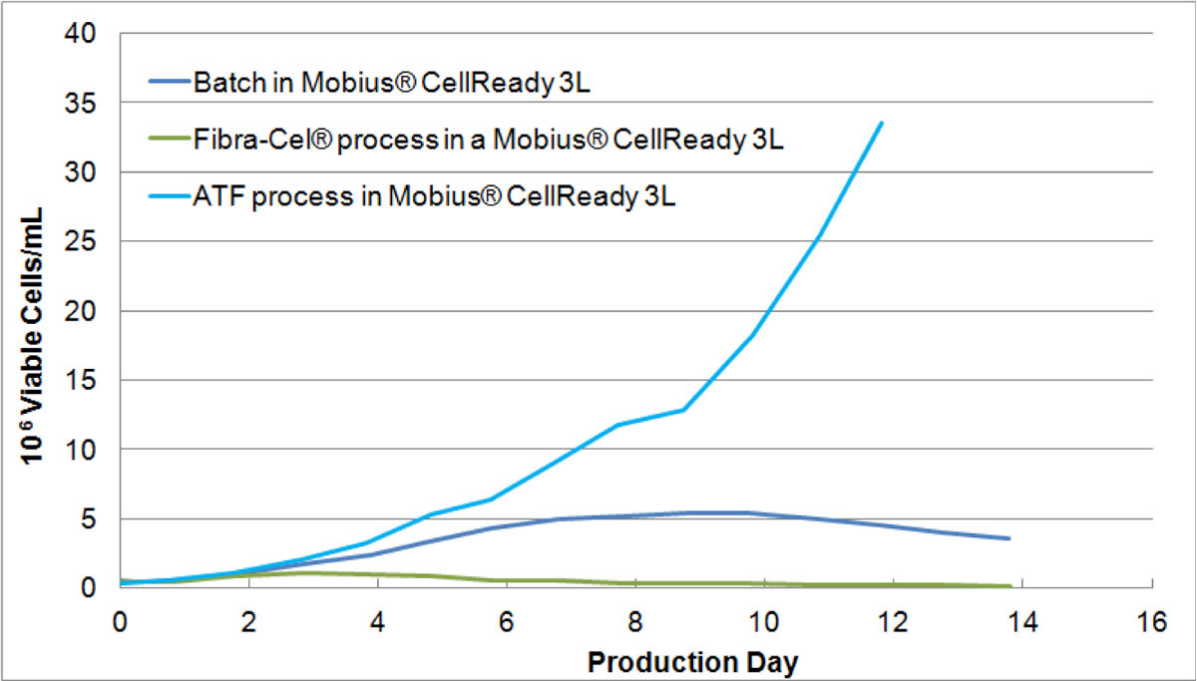
**Viable cell density in suspension in batch and perfusion processes measured in Mobius^® ^CellReady 3L**.

As expected, the cells grew on Fibra-Cel^® ^Disks after 2 days. Thus only a few cells were in suspension from day 3 to day 14 (end of the bioreactor). Regarding the ATF™ technology, a maximum cell density of 33 millions cells/mL was reached (Figure 1).

The glucose concentration was well maintained between 5 and 6,5 g/L while the lactate was not above 1.5 g/L in perfusion bioreactors. A steady state was maintained over several days. The global productivity of each process mode was calculated and compared to the batch one. The perfusion technologies increased the mAb quantity obtained compared to a batch mode. The ATF™ technology increased the final mAb titer by 2.9 fold and the Fibra-Cel^® ^technology increased the mAb quantity by 1.2 fold (Table 1).

**Table 1:** Global productivity, Host cell Proteins, High Molecular Weight and Low Molecular Weight contents in perfusion processes compared to batch ones reached in Mobius^® ^CellReady 3L bioreactor in addition to upstream cost to reach ATF™ mAb quantity, in perfusion processes compared to batch one in Mobius^® ^CellReady Family

	Batch mode	Fibra-Cel^® ^technology	ATF™ technology
Final Titer	100%	121%	290%

Host Cell Proteins	100%	28%	144%

High Molecular Weight	100%	87%	198%

Low Molecular Weight	100%	68%	107%

Upstream cost at 3L GLP Scale	100%	108%	47%

Upstream cost at 50L GLP Scale	100%	175%	84%

Upstream cost at 200L GMP Scale	100%	134%	47%

The quality attributes of the mAb obtained in batch and perfusion modes were also compared. The molecule produced during the perfusion processes was more acid than the ones produced in batch and fed-batch modes. Therefore the mAb produced with Fibra-Cel^® ^and ATF™ technologies in Mobius^® ^CellReady 3L bioreactor could have a higher half-life than the molecule produced in batch and fed-batch modes. Regarding the Host Cell Proteins, Low Molecular Weight and High Molecular Weight overall contents, the ATF™ technology generates more contaminants while the Fibra-Cel^® ^reduces them compared to a batch process (Table 1). Finally, the upstream cost to reach the ATF™ quantity was compared between batch and perfusion processes at different scales. The ATF™ technology can reduce process cost in disposable bioreactors whatever the scale compared to the batch mode while the Fibra-Cel^® ^process cost is higher due to higher medium quantity necessary (Table 1).

## Conclusions

Without any modification of the Mobius^® ^CellReady 3L bioreactor, we were able to demonstrate the compatibility of this single use bioreactor to a mAb perfusion process. Using two different technologies, the overall performances, molecule quality, contaminant level and cost were compared. This study demonstrates the flexibility of existing disposable bioreactors to new bioprocessing technologies.

